# Medicine

**Published:** 1921-06

**Authors:** Cecil Clarke


### Progress of the Medical Sciences.

### MEDICINE.

The knowledge of the **blood chemistry in disease** is becoming of wider application largely as a result of the labours of the American schools of medicine. Since Folin[1] working with 10 c.c. of blood introduced a technique enabling one to estimate all the important non-protein nitrogenous elements of the blood an enormous amount of clinical and pathological work has been done in diseases of the kidney, gout, cardiac failure, and diabetes. In diseases of the kidney, whether primary or associated with cardiac disease, the examination is made mainly with a view to determining the presence or absence of non-nitrogenous waste substances; in diabetes to ascertain the extent of the hyperglycæmia and the state of the alkali reserve or "acidosis." The normal values for each constituent are now well established, although minor differences as to the exact figures still exist. Each normal constituent has, it seems, its own degree of variation, creatinin–N (1–2 mgs. per 100 c.c.) at one end of the scale showing the least variation, with urea–N at the other end showing greater variations (11.5 to 20 mgs. per 100 c.c.). The standard time given for collection of the blood to establish the normal percentages is before breakfast, after a fourteen-hour fast. The upper limit or "threshold value," a term which assumes that excretion is a mechanical effect of an accumulation of the particular substance in question beyond a certain concentration, is given for urea by MacLean[2] as 40 mgs. per 100 c.c. The majority of writers give a figure below 30 mgs.* In excreting the creatinin of the blood the

* E. L. Kennaway[8] gives 15 to 60 mgs. per 100 c.c. as the normal for urea in perfectly healthy subjects. These figures are obtained from normals without reference to the time of obtaining the specimen or the control of diet.

Gettler and St. George,[9] from figures based on 15,000 determinations, define normal limits as follows :—

| | Normal. | Nephritis. | Cardiac Failure. | Diabetes. |
|---|---|---|---|---|
| Number of cases studied .. | — | 600 | 350 | — |
| Non-protein, Nitrogen .. | 25–40 | 40–460 | 35–220 | — |
| Urea–N .. .. .. .. .. | 10–18 | 20–375 | 18–180 | — |
| Creatinin .. .. .. .. .. | 0.1–0.8 | 2–42 | 1.5–12 | — |
| Uric Acid .. .. .. .. .. | 0.5–3 | 3–17 | 2.5–7 | — |
| Sugar .. .. .. .. .. .. | 60–110 | 75–160 | 70–135 | 105–1010 |
| Alkali reserve .. .. .. | 53–80% | 40–75% | 48–75% | 60–70% |

All figures throughout represent milligrams per 100 c.c. blood.

kidney concentrates 100 times, in the case of urea 80 to 100 times, and with uric acid (blood uric acid 2 to 3 mgs.) 20 times. In renal insufficiency uric acid will be the first to accumulate and creatinin the last.

Fortunately for the practical application of these estimations to clinical medicine it is found that there is a general parallelism between the accumulation in the blood of urea–N and the other non-protein N-substances. The former can be estimated with a 3 c.c. quantity of blood sufficiently accurately and without a profound knowledge of chemical technique.[2] The principle of the method depends on the fact that the soya bean contains a specific enzyme (urease) which converts urea quantitatively into ammonium carbonate, but has no effect on any other nitrogenous constituent. There is general agreement that an estimation of the blood urea-N is a satisfactory index of the functional power of the kidney. This estimation has a practical application in conditions of nephritis, cardio-renal disease, and in genito-urinary surgery. Gettler emphasises the fact that the blood analysis should be considered as an adjunct to the clinical investigation. Even so, when all the clinical and laboratory evidence may point to a certain form of nephritis, the post-mortem examination will not necessarily be in agreement with the diagnosis made during life.

In acute nephritis figures of 20 to 40 mgs. indicate that the power to excrete nitrogenous waste is undamaged, while if the figures are high, 100 mgs. or more, the prognosis is serious if this high level remains or tends to increase. Such retention occurs also in cardio-renal disease. Widal, Weill and Vallery-Radot[3] state that subjects with urea-N reaching 100 mgs. without remissions almost always die within two years; moreover, such a figure is indicative of a terminal phase. They note that patients with a figure of 50 to 100 mgs. may not undergo any notable deterioration, but they regard the finding as a valuable warning indication. O'Hare in two cases noted a blood urea figure of 59 mgs. and 50 mgs. respectively, with death three and five years later. The autopsy showed advanced arterio-sclerotic changes in the kidneys.

In the renal deficiency associated with back-pressure effects in prostatic enlargement a figure above 30 mgs. indicates a definite degree of operative risk. Preliminary cystotomy in a favourable case causes a fall in the figure (Dobson[4]).

Myers and Killian[5] consider the blood creatinin figures furnish a more reliable prognosis than any other test. They base their conclusion on an examination of 85 cases who had over 5 mgs. creatinin (some as high as 33.3 mgs.); 80 of the cases died within a few months of the observation. At the same time blood urea-N was estimated, and it is clearly shown that disturbances in the blood urea-N content occur at an earlier stage than in the creatinin-N content. Folin showed that this substance is endogenous, the amount excreted from day to day being a constant for the individual. It can be considered as an index of the amount of active protoplasmic tissue, a great increase meaning a serious impairment of renal tissue.

The chemistry of the blood proteins becomes of very great clinical importance in view of Epstein's work. Although there are sharp differences of opinion as to the correct interpretation of the findings and also as to the correctness of the findings, the practical application of the theory in Epstein's diet in chronic nephritis with œdema has met with success. The normal figures given for the protein content of the serum are 6.5 to 8.5 grm. per 100 c.c. of blood; of this the globulin fraction does not normally exceed 35 per cent., the cholesterol content is 200 mgs. Epstein[6] considers that the œdema in chronic degenerative tubular nephritis is largely due to a disturbance of the blood protein percentage with corresponding alteration of the albumin-globulin ratio and an increase in the amount of lipoids. In an illustrative case the analysis showed protein 4.38 gms., globulin 3.2 gms. or 75.5 per cent. of the protein, cholesterol 840 mgs. Stated briefly, the theory is that the cause of the œdema is the decreased osmotic pressure of the blood resulting from the diminution of the protein content of the blood serum, a condition directly due to the steady loss of large quantities of albumen in the urine. The altered condition of the blood serum and the consequent reduced osmotic pressure favours the absorption and retention of fluid by the tissues. Hence the great œdema and oliguria. It is to be understood that Epstein in no way disputes the factor of deficient chloride elimination as a cause of œdema in chronic diffuse nephritis. The Epstein diet is designed to increase the protein content of th serum, and thus to restore the osmotic pressure of the blood and remove the excessive lipoids.

A further observation of interest is that the œdema fluid has a quite distinctive analysis.* It consists chiefly of water, salt and non-protein nitrogenous substances present in a concentration similar to that found in the blood, with a small amount of protein.

Whatever be the correct explanation of the above facts,

* In one case quoted œdema fluid gave :—

| | Fluid. | Blood. |
|---|---|---|
| Non-protein N .. .. .. .. .. .. | 122 | 114 |
| Urea-N .. .. .. .. .. .. .. | 84 | 60 |
| Uric Acid .. .. .. .. .. .. .. | 3.5 | 2.5 |
| Creatinin .. .. .. .. .. .. .. | 4.5 | 2.1 |

alterations in the dietary based on such findings cause a general improvement and a disappearance of the œdema in some cases. MacLean considers that the increase in the urea content of the blood following on the high protein diet is the cause of the improved renal function, *i.e.* the increased diuresis. Acting on this supposition, MacLean and Russell gave 30 gms. or more of urea daily with undoubted benefit to cases of parenchymatous nephritis with œdema and ascites, maintaining the administration for several weeks when necessary.

Epstein remarks that the increased diuresis must occur via the blood, and that the observed increase in the urea is to be held responsible for the diuresis is undoubtedly a misinterpretation of facts. It is more likely, he considers, that the increased concentration of the urea is the result of a temporary accumulation of this substance in the blood caused by the passage of the œdema fluid from the tissues to the kidneys.

Kahn,[7] on the other hand, examined many different types of kidney disease with a view to verifying the statement that the albumin-globulin ratio is disturbed. He found that the normal ratio 1.5 or 2 : 1 remained practically unchanged both under ordinary conditions and when the patient is fed on a high protein diet.

Gettler and St. George[9] consider that in diabetes the estimation of the blood sugar is more important than that of the urinary sugar; they state, moreover, that hyperglycæmia can be detected a long time before sugar appears in the urine. The blood analysis figures can, as is known, be influenced by diet and more especially in the case of the carbohydrate element. Hyperglycæmia was present in some fifty cases of carbuncles and furunculosis of severe grade. The conditions improved rapidly when the patients were placed on a diet poor in carbohydrate.

**Gastric Function.**—The fractional analysis of the Boas test-meal, introduced by Rehfuss and his co-workers in 1914, has added greatly to our knowledge of the vagaries of gastric secretion and motility in disorders of the stomach. Hitherto most of the facts known have been elicited by the employment of the test-meal of Ewald examined at an interval of an hour later. Correct figures are obtained if the removal of such a specimen is assisted by the employment of Senoran's aspirator. But as is easily demonstrable by the fractional method, a chance sample at the end of an hour may give a result approximately the same in several types of secretory behaviour with varying diagnoses. The estimation of the acidity at quarter-hourly intervals gives a much truer picture of the actual conditions.

There are further obvious advantages. We obtain a knowledge of the amount and character of the resting juice, macroscopic inspection for bile, mucus and blood is possible, and lastly we learn the rate of emptying.

Ryle and Bennett[10] working with a gruel test-meal have established normal standards—results based on the investigation of one hundred healthy medical students. The normal resting juice shows wide variations in amount. Figures above 50 c.c. are unusual, fully 10 per cent. of normals show no resting juice. The free acidity with Töpfer's reagent as indicator varies between 0 and 20. Rehfuss and Hawk give much higher figures with an average of 52 c.c., total acidity 30 and free acidity 18. Bile is present in more than 50 per cent. and trypsin in practically all specimens.

At least four types of normal curves can be recognised: (1) An average normal group (75 per cent.), total acidity rising to 30 (.109 per cent. HCl), emptying in 2½ hours; (2) hyperchlorhydria (12 per cent.), acidity rising to 70 (.255 per cent. HCl); (3) hypochlorhydria and achlorhydria (10 to 12 per cent.); (4) rapid emptying in about an hour, with average acidity.

In the investigation of unselected cases of dyspepsia the most striking differences are seen in an increase in number of "hyposecretory" curves—about 25 per cent., and of these about one-third show complete achlorhydria throughout the cycle of gastric digestion. In about 30–35 per cent. there was definite hypersecretion. In 30–40 per cent. the readings fell within normal limits, though the curves might show abnormal characters.

The clinical associations of these two conditions are of interest. Apart from the traditional finding of absence of free hydrochloric in carcinoma of stomach and pernicious anæmia achlorhydria or marked hypochlorhydria has been very constantly demonstrated in cases of acne rosacea.

Barber obtained strikingly successful results in this disorder by giving acid hydrochloric dil. B.P. in doses of 30 minims and upwards with meals. Of twelve of these cases investigated by the fractional test meal there was complete achlorhydria or an extreme degree of hypochlorhydria in seven and a delayed rise in acidity in one.

The hypersecretory curve[11] was obtained in most instances from patients with history, symptoms and sometimes X-ray findings pointing to a diagnosis of duodenal or pyloric ulcer. Similar curves have, however, been obtained where the above conditions were not suspected.

One other observation is of considerable importance. Definitely proven cases of lesser curvature ulcer have shown normal or hyposecretory types of curve and in a few instances almost complete achlorhydria.

The chief points of the hypersecretory curve can be summarised as follows: a copious clear resting secretion, 60 to 100 c.c. or more of a high acidity (50 to 80); a steadily ascending acidity, average 73, the summit of the curve coinciding with emptying; the rate of emptying between two and two and three-quarter hours.

A smaller group showed "duodenal hurry," emptying in three-quarters to one and a half hours.

Rehfuss and Hawk,[12, 13] from a study of 842 normals, bring forward certain facts and considerations which enable one to realise the undesirability of assigning to any given secretory curve a pathological interpretation without a very full understanding of the normal secretory and motor response. They recognise three normal secretory responses, hypersecretion (40 per cent.), isosecretion (30 per cent.) and hyposecretion (30 per cent.); similarly three motor responses, fast, normal and slow. The digestive phase is made up of (1) the psychic secretion, total acidity 97.6 (0.35 per cent.), (2) the chemical secretion.

The latter begins very early in digestion; it is more persistent than the former, lasting throughout digestion and frequently continuing after the stomach is empty. In health the quantity secreted with a mixed meal is over 200 c.c. The type of meal used for the test influences the secretory response. With foods, notably meat and fish, acidities over 100 were quite common (42 per cent.); for meat the average figure for total acidity was 120, for eggs 80, and for vegetables 70. Defining the normal acidity as 70 to 80 (0.25 per cent. to 0.28 per cent.), they found that this figure was exceeded in 40 per cent. of normals.

While recognising "hyperacidity" as a definite clinical entity, they point out that a demonstrable titratable acidity does not of necessity exist. In fact, in some patients with definite symptoms relieved by alkalies hypoacidity was present. On the other hand, no acid figures found in disease have exceeded the figures which obtain sometimes in health. It seems clear that the line of demarcation between the end of normal findings and the beginning of pathological manifestations is exceedingly difficult to define. The figures given for ulcer are in general agreement with the findings of the English workers—38 per cent. showed hyperacidity and 30 per cent. low acidities.

Criticism of the fractional test is directed mainly against the chemical methods employed in the analysis. Manifestly it is impossible to make a complete analysis of twelve specimens. The advocates of the detailed examination of the chlorides point out the necessity in gastric analysis of ascertaining the amount of mineral chlorides. It is established that a characteristic finding in gastric carcinoma is the relative increase of the mineral chlorides. This also occurs when for any reason there is regurgitation of intestinal contents into the stomach, the physiologically active hydrochloric acid being neutralised in part and sodium chloride formed. The conditions under which this occurs are stated to be chronic gastric ulcer, duodenal ulcer, gall-stones, and of course gastro-enterostomy.

All observers, however, agree that either method should be supplemental to a detailed clinical examination. The fractional meal gives very definite information in cases of pyloric stenosis, whether due to carcinoma or ulcer, as does the one-hour test. The study of the cases should be completed by an examination of the fæces for occult blood.

The method offers wide possibilities for physiological research as to the effects of the emotions, action of drugs, gastric secretion, and motility in health and disease.

REFERENCES.

[1] Folin, *J. Biol. Chem.*, 1919, xxxviii., 81.
[2] Maclean and Russell, *Lancet*, 1920, i., 1305.
[3] Widal, *Med. Press and Circ.*, 1919, ii., 323.
[4] Dobson, *Brit. M. J.*, 1921, i. 289.
[5] Myers and Killian, *Am. J. M. Sc.*, 1919, clvii. 674.
[6] Epstein, *Med. Clin. N. Amer.*, 1920, iv. No. 1.
[7] Kahn, *Arch. Int. Med.*, 1920, p. 112.
[8] Kennaway, *Brit. J. Exper. Pathol.*, June, 1920.
[9] Gettler, *J. Am. M. Ass.*, 1918, lxxi. 2033.
[10] *Lancet*, 1920, ii. 490.
[11] Ryle, *Guy's Hosp. Rep.*, 1921, lxxi. 43.
[12] *J. Am. M. Ass.*, lxxv. 449.
[13] *Am. J. M. Sc.*, 1920, clx. 428.

CECIL CLARKE.

